# From aging biology to cardiac biotechnology: emerging platforms for modeling cardiac aging

**DOI:** 10.1172/jci.insight.203447

**Published:** 2026-08-10

**Authors:** Kritika Chaddha, Mabel Bartlett, Tzahi Cohen-Karni, Aditi Gurkar

**Affiliations:** 1 Aging Institute, Department of Medicine, University of Pittsburgh, Pittsburgh, Pennsylvania, USA.; 2 Biomedical Engineering and; 3 Materials Science and Engineering, Carnegie Mellon University, Pittsburgh, Pennsylvania, USA.; 4 Division of Geriatric Medicine, Department of Medicine, University of Pittsburgh, Pittsburgh, Pennsylvania, USA.

## Abstract

Aging is a major contributor to cardiovascular disease and mortality in older adults. Yet most preclinical and experimental cardiac studies fail to account for age as a primary biological variable, leaving a critical gap in our understanding of how aging contributes to disease progression. Bridging this gap requires integrating aging biology, cardiac pathophysiology, and cutting-edge biotechnology to uncover the mechanisms underlying age-related cardiac dysfunction. We offer a new approach methodologies (NAMs) perspective on how emerging bioengineering strategies may reshape the study of cardiac aging by enabling multidimensional monitoring of cardiac function, aging trajectories, and therapeutic responses. To capture this complexity, we propose the A×G×E×D framework, where A stands for age, G for genetics, E for environment, and D for drug exposure, as a multidimensional lens for understanding how these factors converge to determine cardiac vulnerability during aging. We highlight the integration of long-term cardiac microtissues with advancements in biotechnology to model age. This Perspective opens new frontiers for understanding how A×G×E×D interactions manifest at the molecular, cellular, and electrophysiological levels and for designing responsive, personalized interventions that align with each individual’s evolving physiology. By developing robust bioengineered platforms that recapitulate human cardiac aging, we can advance toward precision geromedicine for cardiovascular health.

## Introduction

Cardiac aging represents a fundamental challenge in modern medicine, as age-related changes in cardiac structure and function underlie the majority of cardiovascular diseases in older adults (1). The aging heart exhibits progressive deterioration, including cardiomyocyte (CM) hypertrophy, increased fibrosis, impaired diastolic function, reduced contractile reserve, and electrical remodeling, all of which increase vulnerability to heart failure, arrhythmias, and ischemic disease (2, 3). Aging interacts with genetic predisposition (4), environmental exposures (5), and pharmacological interventions to determine cardiovascular outcomes, requiring models that recapitulate human cardiac aging with high fidelity (6).

Small animal models, particularly mice, have provided valuable insights into cardiac biology; however, fundamental differences in heart rate (500–600 bpm vs. 60–100 bpm in humans), action potential duration, ion channel expression, and metabolic characteristics limit translational relevance (7–12). Inbred mouse strains also fail to capture the genetic diversity and environmental heterogeneity of human populations (13). The FDA Modernization Act 3.0 and recent NIH initiatives promote new approach methodologies (NAMs) (14): alternative methods to traditional animal testing that incorporate human-relevant models and advanced technologies, representing a paradigm shift toward human-centric, mechanistically informed functional platforms (15). These platforms capture complex cardiac behaviors, including electrophysiological activity, action potential dynamics, conduction velocity, contractile force, and emergent rhythmic properties, such as heart rate variability, moving beyond endpoint toxicity toward continuous, multiparametric readouts of living cardiac tissue (16, 17).

Human induced pluripotent stem cell–derived (hiPSC-derived) cardiac models offer unprecedented opportunities to study cardiac aging in human genetic backgrounds (18). hiPSCs are amenable to genome editing (19), which offers a powerful approach to investigate the effect of specific genetic polymorphisms on cardiac aging in isogenic backgrounds by eliminating confounding effects of genetic variation and enabling precise dissection of genotype-phenotype relationships (20). Combined with 3D organoids, organ-on-chip systems, and integrated bioelectronics, NAMs enable long-term culture, functional monitoring, and mechanistic interrogation of aging processes (21). This Perspective explores how NAMs can model cardiac aging, emphasizing (a) the A×G×E×D framework for understanding multifactorial axes, (b) strategies to simulate biological and chronological aging, and (c) advanced platforms for longitudinal assessment of aging trajectories.

## A multidimensional framework for cardiac aging: the A×G×E×D model

**Figure 1:**
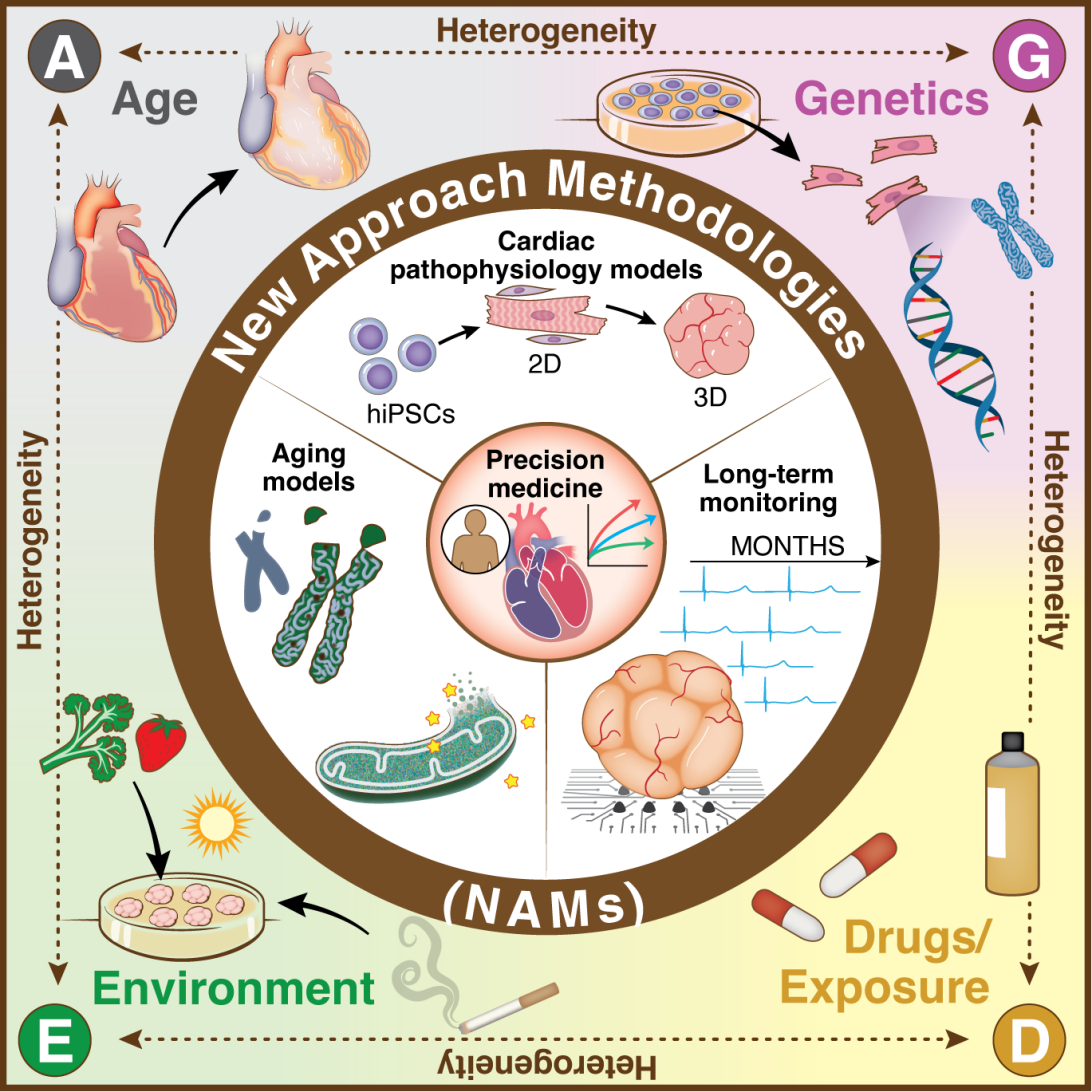
Integrating age, genetics, environment, and drug exposure through new approach methodologies to model cardiac pathophysiology and heterogeneity. New approach methodologies (NAMs) have the potential to allow a comprehensive understanding of cardiac aging and disease by integrating multiple axes — age (A), genetics (G), environment (E), and drug exposure (D). By combining hiPSC-derived cardiac constructs and advanced monitoring technologies, the NAMs framework provides a multidimensional view of how A×G×E×D interactions govern cardiac health and therapeutic response, driving innovation toward targeted, patient-specific interventions (precision medicine).

Cardiac aging arises through the interplay of multiple biological axes that determine cardiac vulnerability and resilience. We define this as the A×G×E×D framework, reflecting the convergence of four axes: age (A), genetics (G), environment (E), and drug (D) exposure (Figure 1).

Age encompasses progressive deterioration of cardiac resilience throughout the lifespan, impairing mitochondrial competency, proteostasis, DNA repair, and inflammatory regulation, diminishing the myocardium’s ability to withstand metabolic, oxidative, and mechanical stress (22). The rate of functional decline varies substantially between individuals, reflecting interactions with other axes.

Genetics, including SNPs, gene expression profiles, and epigenetic modifications, defines intrinsic susceptibility by governing mitochondrial robustness, drug metabolism, redox homeostasis, and sarcomeric stability (23). For example, variants in the genes encoding myosin binding protein C3 (MYBPC3) (24) and titin (TTN) usually remain clinically silent in youth but manifest as cardiomyopathy or hemodynamic stress with age (25). Polygenic risk scores for coronary artery disease show stronger predictive power in older cohorts, suggesting that age amplifies genetic vulnerability (A×G) (26).

Environment encompasses diet, physical activity, toxin exposure, and comorbidities such as diabetes, obesity, and hypertension. Sedentary behavior, for example, compounds age-related arterial stiffness more dramatically in older adults (A×E), disrupting metabolic flux, cardiac homeostasis, and inflammatory balance (27).

Drug/exposure reflects the dose, duration, and mechanisms of pharmacological agents, including therapeutics, pollutants, and occupational exposures, that introduce cardiac stress. Common drugs, such as NSAIDs and fluoroquinolones, carry disproportionately higher cardiac risk in aging populations (A×D). NSAIDs, widely prescribed for arthritis and musculoskeletal pain in older adults, inhibit COX-2–derived prostacyclin (PGI_2_) synthesis, shifting the hemostatic balance toward a prothrombotic state that increases the risk of myocardial infarction and stroke, particularly with chronic use (28, 29). Fluoroquinolones, commonly used broad-spectrum antibiotics for urinary and respiratory tract infections, block the cardiac hERG (KCNH2) potassium channel, prolonging the QT interval and predisposing to torsades de pointes while more than doubling the risk of aortic aneurysm or dissection in older patients (30, 31).

A defining strength of NAM platforms is the capacity to operationalize this framework, recognizing that these axes interact synergistically rather than independently. Adverse convergence, even minor perturbations, can trigger cascading electrical abnormalities, structural remodeling, and functional impairment. hiPSC-derived cardiac tissues and 3D organoid platforms are uniquely positioned to simulate these interactions in controlled, human-relevant systems, enabling mechanistic dissection of individual susceptibility, longitudinal disease tracking, and personalized risk stratification strategies beyond the reach of conventional animal models.

## Modeling cardiac aging for clinical impact: a NAM-based framework

**Figure 2:**
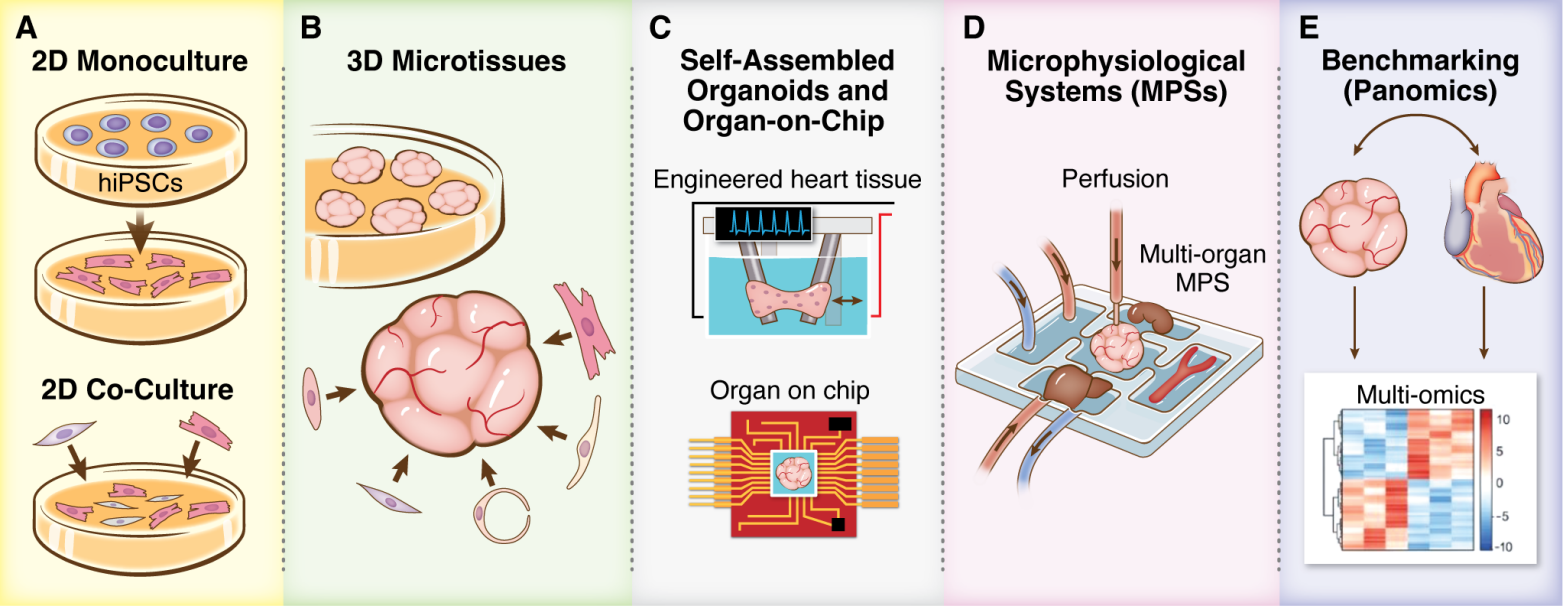
Advancing human cardiac modeling platforms from 2D cultures to complex 3D systems. Schematic overview of model platform progression. (**A**) 2D monoculture and coculture systems. (**B**) 3D microtissues enable multicellular organization. (**C**) Self-assembled organoids/organ-on-chip platforms. (**D**) Microphysiological systems (MPS) enhance physiological relevance with perfusion and multiorgan integration. (**E**) Panomic benchmarking (genomics, transcriptomics, proteomics, metabolomics) ensures model fidelity to human heart capturing A×G×E×D.

Modeling human-relevant cardiac aging demands platforms that capture the integrated A×G×E×D framework. hiPSCs provide the cellular foundation, enabling differentiation into CMs, fibroblasts, endothelial cells, and smooth muscle cells (32), while direct reprogramming (transdifferentiation) via lineage-specific transcription factors offers a complementary route bypassing the pluripotent state (33, 34). 2D monocultures support high-throughput screens, including genome-wide CRISPR/Cas9 screens (35) and high-content imaging studies tracking mitochondrial morphology, calcium dynamics, and contractile function at single-cell resolution (36), though they lack the structural complexity of native myocardium that 3D platforms are now addressing (37) (Figure 2).

3D cardiac organoids and microtissues represent a significant advance in physiological relevance. Self-assembled multicellular human cardiac organoids partially recapitulate human cardiac function and enable tissue maturation and aging beyond what 2D cultures achieve (38–40). Organ-on-chip devices extend this further, culturing tissues within 3D scaffolds under controlled mechanical and electrical stimulation (40–42). Platforms such as Biowire II, which aligns cardiac tissues with field stimulation protocols (43), and PREDICT-96, a high-throughput microtissue array (44), now enable scalable functional readouts of aging phenotypes. Advanced microphysiological systems integrate multiple tissue types with perfusion (45, 46) (e.g., liver and kidney on the same chip), modeling how circulating metabolites, inflammatory factors, and damage signals from distal organs influence tissue function, recapitulating the multiorgan dimension of aging that isolated cardiac models cannot capture. Critically, perfusion architecture, whether unidirectional or recirculating, governs delivery of oxygen, nutrients, hormones, and paracrine signals between compartments, enabling modeling of how systemic metabolic dysfunction, organ crosstalk, and circulating damage-associated signals amplify cardiac aging phenotypes (45, 46). These systems raise a critical question: how do we confirm these platforms truly reflect age-related pathophysiology?

Rigorous benchmarking against primary human cardiac tissue is essential to confirm that these platforms reflect true age-related pathophysiology. Multi-omic datasets from donor hearts spanning transcriptomics, proteomics, metabolomics, and epigenomics provide molecular references for validation, while functional criteria, including contractile force, conduction velocity, calcium handling kinetics, action potential morphology, and stress response capacity, establish physiological benchmarks and define biological clocks that could track in vitro age progression (Figure 2). Once validated, these platforms can become powerful predictive tools to stratify patient-specific drug responses, identify senolytic and cardioprotective candidates before clinical trials, and model age-drug interactions that animal studies systematically miss. Thus, NAMs are positioned as indispensable bridges between cardiac aging research and therapeutic decision-making. Operationally, this translational impact unfolds across three interconnected stages: drug screening, where iPSC-based cardiac microphysiological systems enable high-throughput cardiotoxicity profiling with pharmacological fidelity (47); biomarker discovery, where longitudinal electrophysiological readouts such as action potential duration and arrhythmia incidence serve as validated translational biomarkers bridging preclinical and clinical risk assessment (48); and clinical stratification, where patient-specific iPSC-CM profiling preserves interindividual variation in drug response pathways, enabling genotype-informed risk stratification for precision cardiovascular medicine (49).

## Ability to simulate the passage of time: aging heart in a dish

Understanding cardiac aging requires integrating molecular, cellular, and tissue-level changes that accumulate over time. The 12 interconnected hallmarks — genomic instability, telomere attrition, epigenetic alterations, loss of proteostasis, disabled macroautophagy, deregulated nutrient sensing, mitochondrial dysfunction, cellular senescence, stem cell exhaustion, altered intercellular communication, chronic inflammation, and dysbiosis — provide a useful organizational framework for cataloging biological processes that contribute to cardiac aging. Though how these hallmarks interact (22), generate an effect which predominates in cardiac tissue, and map onto clinical outcomes remains an active area of investigation.

The cascade begins at the genome. DNA damage accumulates in CMs with age (50, 51), activating ATM/ATR-p53 signaling that drives senescence (52) and metabolic rewiring (53), while telomere dysfunction (54) from replicative and oxidative stress compounds this decline. Genomic damage, in turn, reshapes the epigenome through histone modifications, chromatin accessibility changes, and altered chromatin remodeling factors, locking CMs into maladaptive gene-expression states that compromise regenerative and stress response capacity (55). Importantly, whole-genome sequencing (WGS) of single CMs from aged human hearts revealed age-dependent accumulation of somatic mutations, which were predominantly oxidative in origin (54). Future NAM platforms should incorporate strategies to capture this mutational burden, through single-cell WGS of long-term cultured iPSC-CMs or spatially resolved sequencing, to recapitulate the clonal mosaic landscape of the aged myocardium and its functional consequences. Beyond DNA methylation clocks, aging is associated with progressive shifts in histone modification landscapes, including loss of H3K27me3 at developmentally regulated loci, global histone deacetylation, and altered chromatin accessibility, that lock CMs into maladaptive transcriptional states. In NAMs, these changes can be profiled using ATAC-seq to assess chromatin accessibility dynamics, ChIP-seq targeting key histone marks (H3K27me3, H3K4me3), and live epigenetic reporters such as SIRT1-GFP to track deacetylase activity in real time (55). That failure manifests most acutely in mitochondria, where mtDNA damage, impaired electron transport chain (ETC) activity, and elevated ROS lead to adenosine triphosphate (ATP) exhaustion and arrhythmogenesis (56, 57). As they converge with impaired proteostasis and declining autophagy, these changes overwhelm cellular quality control, driving senescence-associated secretory phenotype (SASP) factor release that disrupts paracrine crosstalk among CMs, fibroblasts, and endothelial cells, ultimately attracting immune infiltration and promoting fibrosis (58). Together, these hallmarks form a mechanistic backbone that NAMs should recapitulate to faithfully model age-related cardiac dysfunction.

**Figure 3:**
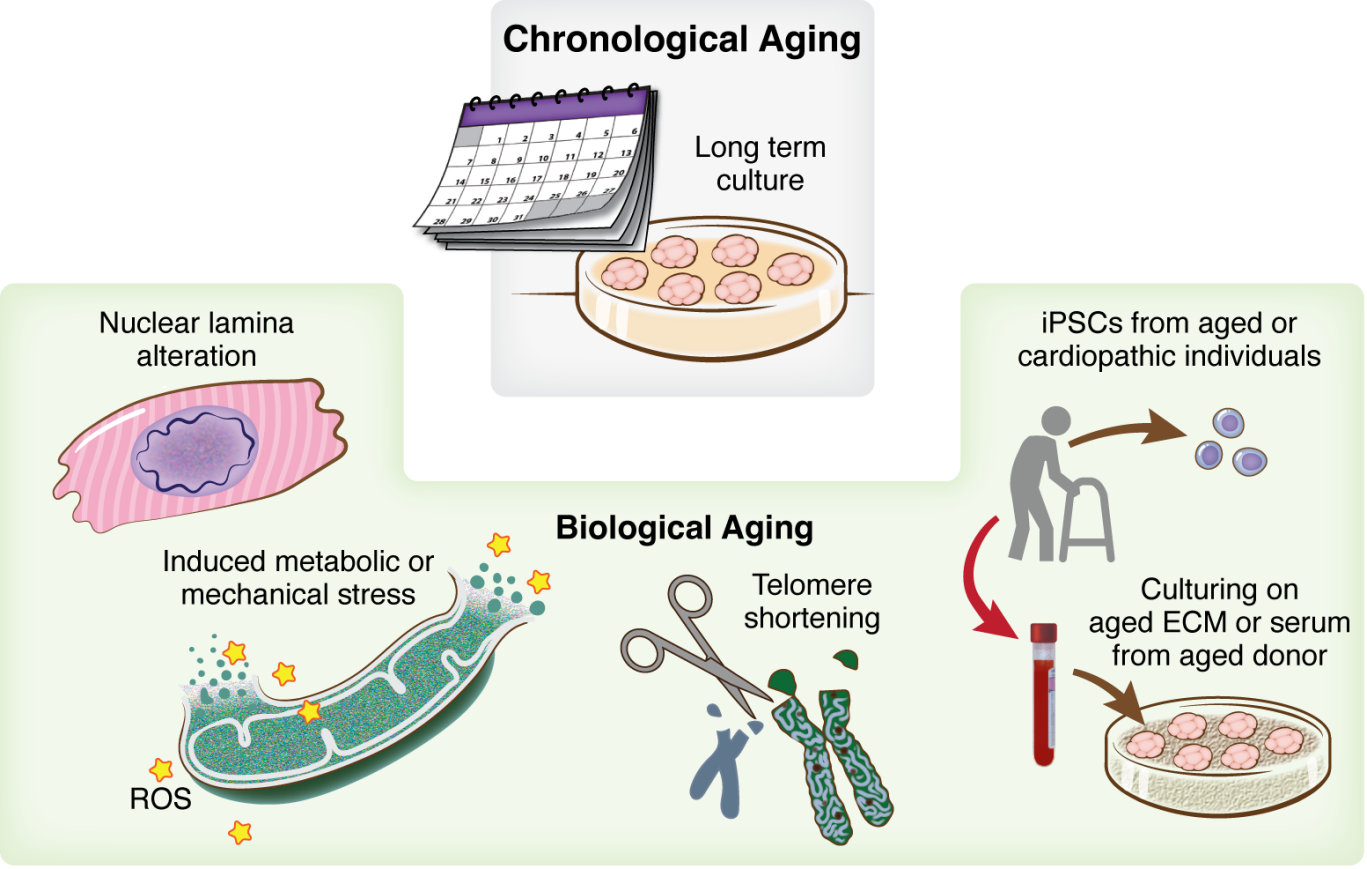
Modeling chronological and biological aging in NAMs. Chronological aging is modeled through long-term culture, allowing time-dependent accumulation of cellular and molecular changes, such as metabolic slowdown, proteostatic decline, and oxidative damage. In contrast, biological aging captures functional decline, induced through stressors, such as telomere shortening, oxidative or mechanical stress, and nuclear lamina alterations. Moreover, biological aging can also be induced by culturing hiPSCs from aged donors, on aged ECM or serum/plasma exposure from older or diseased individuals. These approaches mimic intrinsic and extrinsic damage seen in aging tissue. Together, these complementary strategies reproduce the molecular, metabolic, and structural hallmarks of cardiac aging, enabling investigation of mechanisms driving age-associated cardiac dysfunction and disease susceptibility.

Establishing such NAMs requires careful consideration of both chronological age (passage of time) and biological age (deterioration of functional ability). Cardiac aging has been modeled through long-term culture or exposure to biological stressors, including aged ECM, telomere shortening, nuclear lamina disruption, mechanical and metabolic stress, and aged serum. Aged ECM recapitulates changes in stiffness, composition, and biochemical signaling characteristic of aged hearts (59, 60). Telomere damage can be induced via targeted oxidative stress (61), replicative exhaustion (62), inhibition of telomere shelterin proteins (e.g., TRF2) (54, 63), or CRISPR-based editing (64), while nuclear lamina disruption — as seen through progerin accumulation in Hutchinson-Gilford progeria syndrome (HGPS) — can also accelerate aging phenotypes (65). Patients with HGPS, telomere dysfunction, and other progeroid syndromes, including Werner, Cockayne, and Fanconi anemia, provide iPSC sources for studying biological cardiac aging, with DNA polymerase subunit **γ** (POLG) mutations highlighting the role of mitochondrial genome stability (66–70). Interestingly, iPSC-derived CMs from individuals with end-stage cardiomyopathy are well-established disease models that exhibit accelerated aging phenotypes (71), suggesting that NAM platforms established with these hiPSCs could be a rich resource for simulating age-related cardiac dysfunction and interrogating the molecular mechanisms that couple disease progression to biological aging. Recent literature also suggests that exposure to aged donor serum further recapitulates aging, inducing senescence markers and metabolic shifts in cultured cells (72, 73) (Figure 3, Table 1), suggesting that this strategy is effective to drive biological aging (Table 1).

However, challenges remain in rigorously modeling cardiac aging in vitro. Uncertainty persists about which hallmarks are most appropriate for in situ experiments, how many are needed to reliably track age-related changes, and which endpoint combinations best detect aging across cardiac cell types. Furthermore, heterogeneous aging within the same cardiac unit underscores the need for single-cell resolution approaches. Critically, capturing the progressive and slow-moving nature of cardiac aging demands longitudinal experimental designs, necessitating NAM platforms capable of stable, long-term recording and repeated functional assessment across extended timescales to distinguish transient stress responses from bona fide age-related decline.

## Long-term monitoring platforms that capture dynamic age-related changes

Cardiac tissue functions as a complex bioelectrical system in which electrical signals are intricately regulated by metabolic processes and mitochondrial activity that determine heart rhythm and force of contraction (74). Disruptions from aging, genetic variants, environmental stressors, or drug exposures impair excitation-contraction coupling and drive progressive electrical instability. Conventional in vitro models that rely on brief electrophysiological snapshots are insufficient for capturing long-term aging effects; therefore, technologies capable of probing dynamic changes over extended timeframes are essential within the A×G×E×D framework. Advances in multielectrode arrays (MEAs) (75), voltage-sensitive dyes (76), optogenetic sensors (77), and genetically encoded calcium biosensors (78, 79), are enhancing our ability to study cumulative cardiac aging effects, revealing information about ion channel dynamics, intercellular communication, and single-cell activity across time. Below we discuss the biology underlying specific platforms and the biotechnology needed.

### High spatial resolution.

Cardiac tissue is inherently heterogeneous, with variable gene expression and metabolic states producing nonuniform responses to aging (80). Spatially resolved electrophysiological recordings detect distinct electrical behaviors linking single-cell activity to broader signal propagation, generating isochronal maps that reveal conduction dynamics, including silent zones, directional shifts, and regional amplitude changes, critical for identifying disruptions in cell-cell communication and structural remodeling within aged tissue (81).

**Figure 4:**
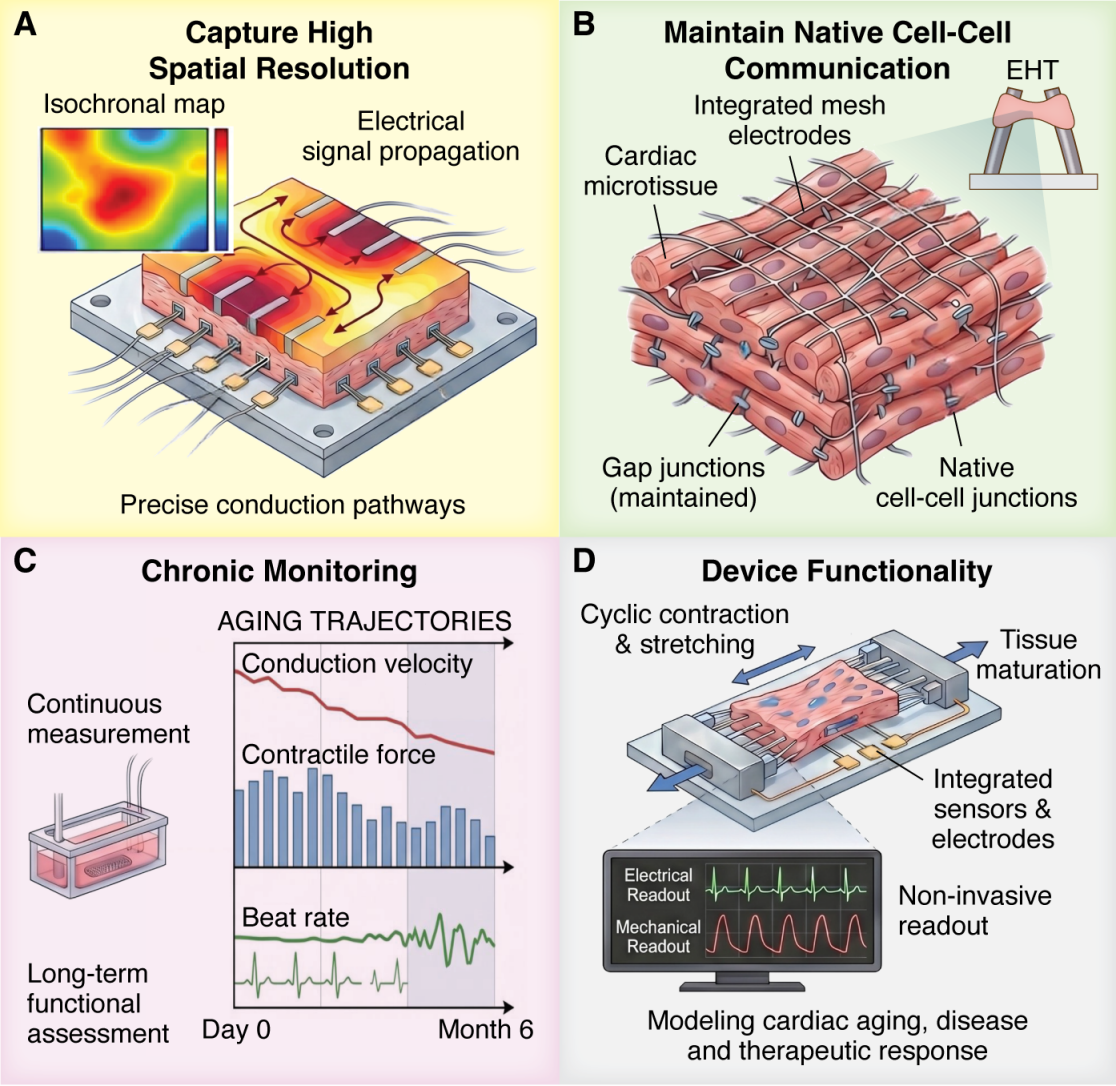
Functional features of cardiac NAMs for high-resolution and long-term functional assessment. (**A**) Capture high spatial resolution. Bioengineered devices should be designed to enable high-spatial-resolution mapping of electrical activity, allowing precise visualization of conduction pathways, signal propagation, and local heterogeneity within cardiac tissues. (**B**) Maintain native cell-cell communication. Integrated mesh electrodes can be embedded within the cardiac microtissues to maintain native cell-cell junctions and tissue architecture, ensuring physiological signal transmission and mechanical integrity. (**C**) Chronic monitoring. Systems designed for chronic monitoring can enable continuous measurement of conduction velocity**,** contractile force, and beat rate over extended culture periods spanning several months. (**D**) Device functionality. Platforms that allow dynamic mechanical stimulation, including cyclic contraction and stretching, which closely mimics the mechanical environment of the native myocardium and promotes tissue maturation would enable a highly rigorous and reproducible NAM. Combined with integrated sensors and electrodes, these devices can provide a noninvasive, high-fidelity readout of electrical and mechanical performance, offering a powerful tool for modeling cardiac aging, disease, and therapeutic response in vitro.

Electrode sizes of 0.1–1 mm and spacing of 0.1–3 mm are generally sufficient to capture localized aging phenotypes and tissue-wide activity (82, 83). Surface-mounted devices support surface-level reconstructions, while embedded devices enable internal recordings of extracellular field potentials (84). For engineered cardiac tissues, embedding 128+ electrodes allows comprehensive 3D mapping (85–88), and future high-density platforms should support electrode placement at 250 to 500 μm intervals (89, 90), while minimizing device footprint to preserve physiological relevance (91) (Figure 4).

### Minimal interference with cell-cell junctions.

Physiological heart function depends on precise electrical and mechanical coupling between CMs (92), mediated by desmosomes, integrins, and cadherins and synchronized via gap junctions at intercalated discs (93, 94). Aging compounds this challenge through structural remodeling, fibrosis, myocyte hypertrophy, and reduced cellular density. Bioelectronic devices must therefore be biocompatible not just in materials but in their capacity to preserve these cellular interactions (95).

Electrodes of 10–20 μm diameter and polymer-encapsulated traces under 10 μm in width and thickness minimize device footprint between adjacent cells (96, 97). Soft, flexible materials enable closer mimicry of the native cellular environment (98), allowing bioelectronic systems to function as biomimetic scaffolds rather than foreign implants, promoting long-term biocompatibility and stable chronic interfacing with the aging heart (Figure 4).

### Preserving device functionality during cardiac tissue contraction.

Reliable long-term recordings require electrode materials and insulating layers to retain structural integrity across tens of thousands of contraction cycles. This challenge is amplified in aging models, where altered tissue stiffness and irregular contraction patterns impose nonuniform mechanical loads that accelerate device fatigue.

Kirigami-inspired structures allow devices to stretch biaxially while keeping material stress below elastic limits (90, 99, 100). Intrinsically stretchable materials and serpentine metal traces offer an alternative, though often at the cost of resolution (101, 102). Kirigami-based devices fabricated via high-resolution photolithography accommodate microscale features needed for precise tissue interfacing (103), offering a promising combination of mechanical resilience and fabrication precision for chronic monitoring over weeks to months (104).

### Tissue monitoring over time (with age).

As next-generation bioelectronics advance, continuous monitoring capabilities within electrophysiological recording platforms emerge as a critical need. Rather than relying on isolated snapshots, continuous monitoring provides a temporal map of aging progression, capturing subtle early-stage changes that might otherwise go undetected (105), which are particularly important when the timing of pathological changes is unknown. In aging cardiac tissue, electrical alterations may emerge gradually or sporadically, and without ongoing monitoring, critical onset of dysfunction may be missed entirely. This is illustrated by doxorubicin-induced cardiotoxicity, where progressive mitochondrial dysfunction and contractile decline are only detectable days after initial exposure (106) and by age-related calcium handling. An important emerging consideration is that acute drug responses frequently diverge from long-term outcomes: studies have demonstrated that 24- to 48-hour drug exposures fail to capture clinically relevant cardiotoxic phenotypes that only emerge over weeks of treatment, underscoring the critical need for extended monitoring windows within the A×G×E×D framework. Once the timescale of interest is identified, periodic sampling protocols, such as recording several minutes every hour or day, can balance data capture with system longevity. A complement to electrophysiology, intermittent fluorescent imaging uses genetically encoded reporters, including calcium indicators such as GCaMP, metabolic sensors such as SoNar (107), and senescence markers (108–110), enables longitudinal molecular readouts that can be correlated with functional electrophysiological outputs without disrupting tissue culture.

Despite progress in long-term electrophysiological recordings (111), most existing studies have been confined to 2D models (112) and short timeframes. A critical gap remains: no existing framework has demonstrated sustained electrical monitoring of 3D cardiac tissue within a fully controlled incubator environment, where maintaining both tissue and electronics in a single stable system reduces risks of thermal stress, physical disruption, or device degradation. Practical implementation would involve integrating perfusion systems alongside electronics capable of routing signals outside the incubator without compromising signal quality. Chronic recordings from 3D cardiac models offer detailed views of how electrophysiological changes unfold in response to intrinsic aging and extrinsic stressors (113, 114), enabling identification of early deviations in electrical patterns that may precede overt dysfunction, ultimately guiding preventative interventions and, in the context of personalized medicine, tailoring treatment to an individual’s unique biological aging profile. Notably, incorporating vascular-like perfusion dynamics, including physiological shear stress, oxygen and nutrient delivery, and endothelial cell–CM coupling is critical for long-term engineered cardiac tissues, as the absence of perfusion induces metabolic stress, inflammatory signaling, and electrophysiological instability that confound aging phenotypes, whereas dynamic flow restores near-adult contractile force, conduction velocity, and metabolic maturation (115, 116) (Figure 4).

### Integrate hallmarks of aging with tissue function.

Traditional aging models have largely focused on single stressors, such as DNA damage or mitochondrial dysfunction. However, aging in vivo is driven by simultaneous and nonlinear activation of multiple hallmarks shaped by intrinsic and environmental factors. Combinatorial stressor models that combine genotoxic stress, mitochondrial uncouplers, and inflammatory cytokines more accurately simulate age-related cardiac dysfunction than single-pathway approaches. Applying these models in iPSC-derived tissues or organoids generates rich, high-dimensional datasets that, when paired with artificial intelligence (AI) and machine learning (ML) (117), can reveal hallmark interactions and support so-called “intervention stacking” strategies that target multiple hallmarks in sequence or tandem. Dynamic aging scores integrating hallmark biology with molecular, cellular, and functional metrics are needed to predict disease risk and functional decline across tissues (118).

Multimodal biosensors are central to this shift toward integration. Expanding beyond electrophysiology to fluorescence-based and electrochemical sensors enables real-time monitoring of ATP, ROS, NADH, glucose, and lactate (119–121), while genetically encoded biosensors report on autophagy, redox state, proteasome activity, ER stress, and cellular senescence (122–126) in live cells. A key limitation is that most biosensors are deployed individually rather than multiplexed, preventing simultaneous monitoring of interacting pathways. Future NAM platforms should integrate multiplexed biosensors to capture dynamic hallmark interactions at a systems level. AI and ML are critical enablers: supervised algorithms classify samples by age or disease state, unsupervised models uncover hidden aging subtypes, and time-series models predict transitions between health and disease by detecting early shifts in mitochondrial dynamics or immune activity (127, 128). Coupled with interpretable AI frameworks and trained on interventional datasets, these models identify therapeutic targets and support causal inferencing. Building this technological and analytical infrastructure for real-time, systems-level aging assessment will lay the groundwork for precision geroscience-enabling tailored interventions, extended health span, and informed aging policy (129, 130) (Figure 4).

## Limitations, challenges, and future directions

Despite their transformative potential, current NAMs exhibit critical limitations. Immune cells cannot be overlooked, as chronic inflammation and immune-cardiac crosstalk are central drivers of age-related cardiac dysfunction. Macrophages can be introduced through coculture systems, including iPSC-derived macrophages that preserve donor-specific genetic context, enabling modeling of SASP-mediated recruitment, inflammatory infiltration, and profibrotic signaling. However, most immune cells have finite lifespans in culture, necessitating periodic replenishment or microfluidic perfusion to sustain physiologically relevant immune-cardiac interactions over the timescales required to study aging meaningfully.

Long-term viability beyond 90–120 days remains technically challenging, limiting the ability to model chronic damage accumulation over timeframes that better approximate human aging. Environmental variables, such as oxygen gradients, nutrient availability, and ECM stiffness, are difficult to control in 3D systems, introducing variability that complicates interpretation. These limitations underscore the need for continued technical refinement, multi-omics benchmarking against primary human tissues, and standardized age-scoring systems. Validation across multiple laboratories and integration of computational models will be essential for translating NAM-based discoveries into clinical applications.

Aging is not merely a background variable in cardiac disease, it is a central mechanistic driver shaping how biological stress is sensed, processed, and amplified within the heart. Realizing the full potential of this platform will require integrating vascularized organoids, immunocompetent microtissues, and embedded multimodal bioelectronics capable of continuous metabolic and electrophysiological monitoring, alongside AI-driven pipelines to interpret high-dimensional time-series data and predict inflection points in tissue aging. By deploying the A×G×E×D framework and modeling biological age directly in vitro, NAMs must ultimately evolve toward closed-loop systems that unite sensors, analytics, and interventions, advancing a new era of precision geromedicine where prediction replaces surveillance and early intervention replaces reactive treatment.

## Author contributions

KC and MB reviewed the literature. KC wrote the first draft. All authors revised and contributed to the article. KC and AUG approved the submitted version.

## Conflict of interest

The authors have declared that no conflict of interest exists.

## Funding support

This work is the result of NIH funding, in whole or in part, and is subject to the NIH Public Access Policy. Through acceptance of this federal funding, the NIH has been given a right to make the work publicly available in PubMed Central.

- NIH grants R01HL161106 to AG, KC, MB, and TCK and R01ES035733 and R21 AG086744 to AG.
- American Federation for Aging Research/Hevolution to AG.
- Richard King Mellon Foundation grant.

## Figures and Tables

**Table 1:**
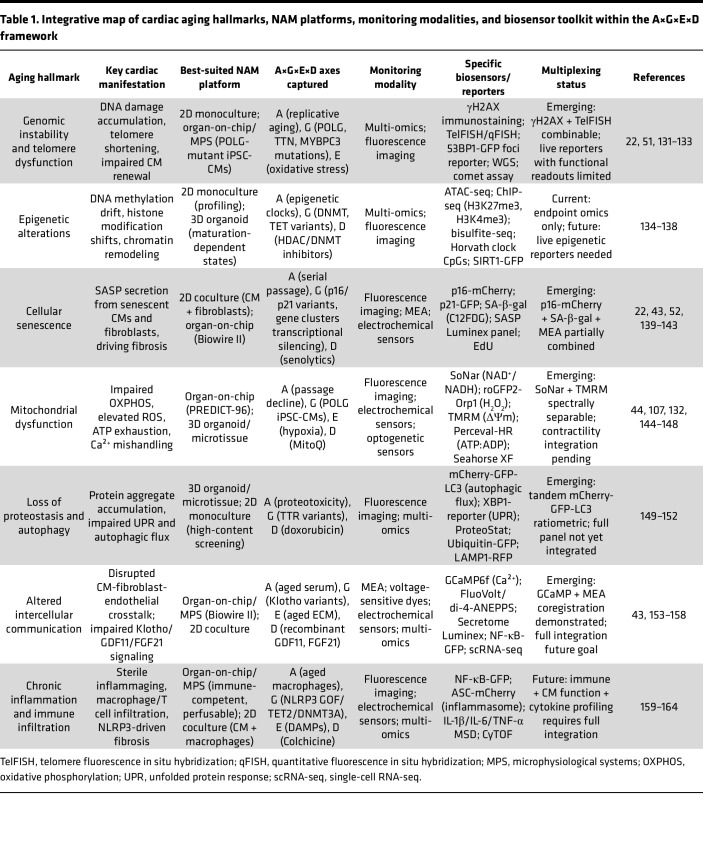
Integrative map of cardiac aging hallmarks, NAM platforms, monitoring modalities, and biosensor toolkit within the A×G×E×D framework

## Version 1 08/10/2026

Electronic publication
